# The ZnO-In_2_O_3_ Oxide System as a Material for Low-Temperature Deposition of Transparent Electrodes

**DOI:** 10.3390/ma14226859

**Published:** 2021-11-14

**Authors:** Akhmed Akhmedov, Aslan Abduev, Eldar Murliev, Abil Asvarov, Arsen Muslimov, Vladimir Kanevsky

**Affiliations:** 1 Institute of Physics, Dagestan Federal Research Center, Russian Academy of Sciences, 367015 Makhachkala, Russia; a-akhmed@mail.ru (A.A.); cht-if-ran@mail.ru (E.M.); 2 Basic Department of Nanotechnology and Microsystem Technology, Academy of Engineering, RUDN University, 117198 Moscow, Russia; a_abduev@mail.ru; 3 Shubnikov Institute of Crystallography, Federal Scientific Research Center Crystallography and Photonics, Russian Academy of Sciences, 119333 Moscow, Russia; amuslimov@mail.ru (A.M.); kanevsky@mail.ru (V.K.)

**Keywords:** transparent conductive oxide (TCO), ZnO, In_2_O_3_, spark plasma sintering (SPS), magnetron sputtering, ceramic target, conductivity, transmittance

## Abstract

The development of optoelectronic devices based on flexible organic substrates substantially decreases the possible process temperatures during all stages of device manufacturing. This makes it urgent to search for new transparent conducting oxide (TCO) materials, cheaper than traditional indium-tin oxide (ITO), for the low-temperature deposition of transparent electrodes, a necessary component of most optoelectronic devices. The article presents the results of a vertically integrated study aimed at the low-temperature production of TCO thin films based on a zinc-indium oxide (ZIO) system with acceptable functional characteristics. First, dense and conducting ceramic targets based on the (100-x) mol% (ZnO) + x mol% (In_2_O_3_) system (x = 0.5, 1.5, 2.5, 5.0, and 10.0) were synthesized by the spark plasma sintering method. The dependences of the microstructure and phase composition of the ZIO ceramic targets on the In_2_O_3_ content have been studied by powder X-ray diffraction, scanning electron microscopy and energy dispersive X-ray spectroscopy methods. Then, a set of ZIO thin films with different Zn/In ratios were obtained on unheated glass substrates by direct current (dc) magnetron sputtering of the sintered targets. Complex studies of microstructure, electrical and optical properties of the deposited films have revealed the presence of an optimal doping level (5 mol% In_2_O_3_) of the ZIO target at which the deposited TCO films, in terms of the combination of their electrical and optical properties, become comparable to the widely used expensive ITO.

## 1. Introduction

TCO-based transparent electrodes are currently an integral component of many optoelectronic devices (displays, light-emitting structures, solar energy converters, biosensors, etc.) [1,2,3,4]. Over the past several decades, the main material for the TCO transparent electrodes formation is binary indium-tin oxide (ITO) due to its high electrical and optical characteristics [5,6]. For all its advantages, the ITO is characterized by one significant drawback—the volume of worldwide extraction of the In, which is the main component of the ITO, no longer meet the needs of unprecedented growing markets of informational displays CuInGaSe_2_ solar cells and light-emitting devices. Currently, the main alternative to ITO is zinc oxide doped with elements of group III of the periodic table Al, Ga and In, which is both inexpensive and widespread. Significant attention has therefore been paid to thin films based on ZnO doped with aluminium (ZAO) and gallium (ZGO) [1,7,8]. This was mainly due to the fact that Al and Ga impurities do not disorder the ZnO lattice as much as indium, which has a large ionic radius. To date, electrical characteristics comparable to ITO have been achieved in Al (or Ga) doped ZnO films deposited at moderate substrate temperatures (up to 300 °C) [9,10]. At the same time, these transparent conductive films deposited at low substrate temperatures (≤100 °C) usually have poor crystallinity and are thus characterized by temporal and temperature unstable electrical properties, which significantly limits their use in flexible electronic devices [11]. In light of this, In has become an attractive n-type dopant for ZnO because it is less reactive and has greater oxidation-resistance [12,13].

This work presents the results of optimization of the composition of sputtered targets for the formation of transparent conducting films based on a zinc-indium oxide (ZIO) system by the direct current (dc) magnetron sputtering method, which is widely used in the mass production of microelectronic devices [14]. At that, the content of the In_2_O_3_ impurity phase in the targets varied in a wide range from 0.5 to 10 mol%. The final goal of this work was to optimize the composition of the film for the problem of low-temperature deposition of transparent conductive ZnO-based thin films with acceptable functional performances.

## 2. Materials and Methods

ZIO thin films were deposited by the dc magnetron sputtering technique using sintered targets. The detailed route for ceramic target preparation is as follows. Submicron powders of zinc (II) oxide (99.95% purity) and indium (III) sesquioxide (99.99% purity) were used as starting reagents. Weighed portions of five mixtures of oxide powders of the following compositions were prepared: (100-x) mol% (ZnO) + x mol% (In_2_O_3_), where x = 0.5, 1.5, 2.5, 5.0 and 10.0.

After 8-h of dry mixing in a ball mill, the prepared powder mixtures were used to form ceramic disc targets with a diameter of 51 mm and a thickness of 4 mm by the spark plasma sintering (SPS) technique [15] under the following technological conditions: residual air pressure in the SPS chamber—0.1 Pa, pressing pressure—25 MPa, SPS temperature—950 °C, SPS duration—3 min, rate of reaching the SPS temperature—60 °C/min, and cooling for 70 min under vacuum with no pressure. After a rubdown, the sintered ceramic discs were examined by powder X-ray diffraction (XRD, PANalytical X’PERT PRO MPD diffractometer with CuKα radiation, Malvern Panalytical B.V., Almelo, Netherlands), scanning electron microscopy (SEM, FEI Scios dual-beam electron microscope, Thermo Fisher Scientific, Waltham, MA, USA) and energy dispersive X-ray spectroscopy (EDX). Additionally, their physical density and electrical resistance was measured by the Archimedes method (balances OHAUS Adventurer™ Analytical AX 124 (OHAUS^®^, Parsippany, NJ, USA) equipped by density determination kit) and the four-probe method (IUS-3, VNIIEM, Moscow, Russia), respectively.

Furthermore, the sintered ceramics were used as targets for dc magnetron sputtering. ZIO thin films were obtained on borosilicate glass substrates with dimensions of 25 × 25 × 1.1 mm, using a magnetron sputtering setup equipped with a drum-type substrate holder [16]. Before the start of each deposition procedure, the vacuum chamber was evacuated to a residual pressure of 2 × 10^−4^ Pa. Sputtering was carried out in Ar working gas at a pressure of 0.3 Pa in the current stabilization mode (*I* = 130 mA). The rotation rate of the drum substrate holder was 10 rpm, and the minimum distance from the target to the passing substrate was 100 mm. The duration of the deposition process is 180 min. It should be noted that during depositions no infrared heating of the substrates was performed. Additional measurements showed that the substrate surface was only slightly heated to temperatures not exceeding 50 °C due to the charged particle bombardment and discharge plasma radiation.

The surface morphology, growth microstructure and elemental composition of the deposited film samples were investigated by using the FEI Scios dual-beam electron microscope. Film thickness determinations were performed by using cross-sectional SEM views in the locally ion etched areas of films. The electrical properties were measured by using the four-probe technique. Optical transmittance spectra were obtained by a UV-3600 optical spectrophotometer (Shimadzu, Tokio, Japan) in the wavelength range of 300–1100 nm. All of the electrical and optical measurements were carried out at room temperature.

## 3. Results and Discussion

**Figure 1:**
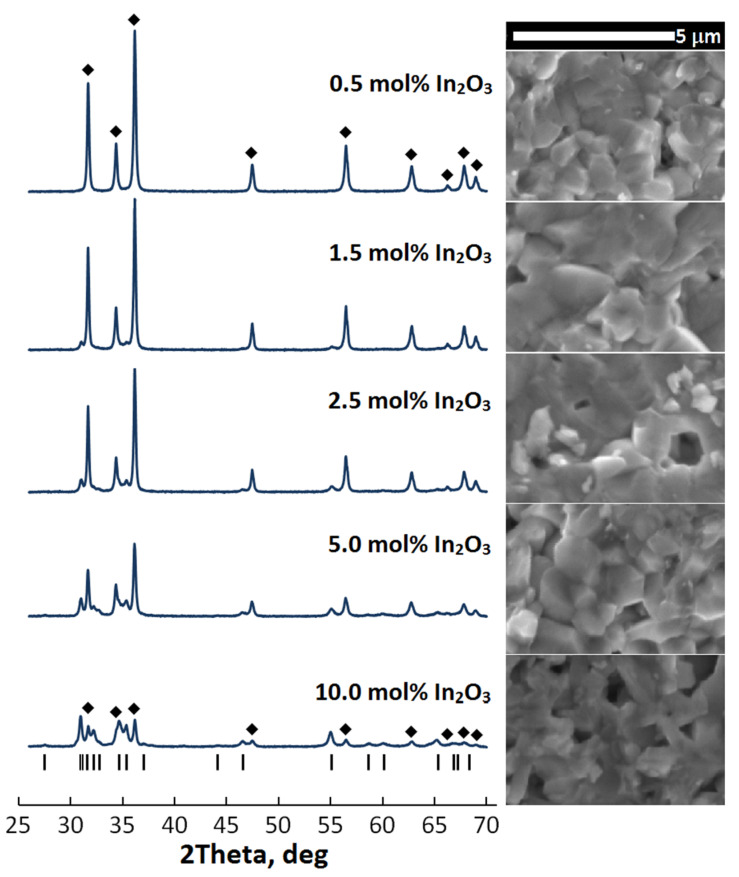
XRD spectra and SEM micrographs of ceramic samples with different In_2_O_3_ content. In the spectra, the peaks corresponding to the hexagonal ZnO phase are marked with the symbol ♦, and the positions of the most intense reflections of the secondary hexagonal In_2_Zn_5_O_8_ phase are marked with black vertical lines.

Figure 1 presents XRD and SEM data for the sintered ceramic samples. According to the XRD data, when 0.5 mol% In_2_O_3_ is added to the initial mixture, the resulting ceramics is a monophase system; in the XRD spectrum there are only reflections corresponding to the hexagonal ZnO phase with a wurtzite structure (JCPDS 00-036-1451). The absence of other reflections in the spectrum may be due to both the insensitivity of the XRD method to small amounts of impurity phases [17] and the complete incorporation of indium into the ZnO crystal lattice without the formation of a significant number of defects such as inverted boundaries passing along the basal {00l} planes of the ZnO crystal lattice [18]. In the XRD spectrum of ceramics with 1.5 mol% In_2_O_3_, insignificant peaks of a secondary phase appear, and with a further increase in the molar fraction of In_2_O_3_ in the system, suppression of reflections of the ZnO phase and an increase in the intensity of numerous reflections corresponding to the secondary phase are observed. Additionally, Figure S1 in the Supplementary Materials (SM) details the XRD spectrum of the sintered ceramics with the maximum content In_2_O_3_ in the initial oxide mixture.

The identification of the forming secondary phase using the PDF ICDD database showed that the observed peaks correspond to the compound In_2_Zn_5_O_8_ (JCPDS 01-089-8974). It was noted recently that in the ZnO—In_2_O_3_ binary system, along with zinc and indium oxides, the most stable compound from the homologous series In_2_Zn_k_O_k+3_ (k = 3–7, 9, 11, 13 b 15) is In_2_Zn_5_O_8_ at sintering temperatures up to 1100 °C [19].

The introduction of significant amounts of indium sesquioxide into ZnO also affects the microstructure of the ceramic. From the micrographs shown in Figure 1, it can be seen that in samples with the In_2_O_3_ content of up to 5.0 mol%, most of the grains havean isotropic form, while in ceramics with a high impurity content, the average grain size decreases and a significant number of anisotropic grains appear among them. Anisotropic lamellar form of grains is characteristic of homologous compounds M_2_Zn_k_O_k+3_ (where M is Ga, In, Fe, etc.) [20]. Additionally, the SEM studies revealed the presence of residual porosity in the sintered ceramics. Measurement of the physical density of the ceramics showed that their density varies without any regularity within 93–96% of the calculated values of the theoretical density for the binary system ZnO–In_2_O_3_. The absence of the regularity can be explained by the fact that the binary system after SPS treatment, as a result, is not a simple mixture of two starting oxides. However, EDX measurements confirmed the preservation of the Zn/In ratio in the sintered ceramic specimens relative to the initial one in the powder mixtures, which confirms the fact that none of the metal components predominantly volatilizes during the rapid SPS treatment under the above conditions.

Evaluation of the sheet resistance *R_S_* of ceramic samples of the same thickness showed that the *R_S_* smoothly decreases from 11.2 × 10^−3^ to 8.3 × 10^−3^ Ω/sq with an increase in the In_2_O_3_ content from 0.5 to 5.0 mol%, and then increases slightly to 9.1 × 10^−3^ Ω/sq at 10.0 mol% In_2_O_3_. The observed low values of the electrical resistance are due not only to the mutual alloying of oxides in the binary system with the formation of electrically conductive phases, but also to the fact that a significant oxygen deficiency could form in the ceramic body during SPS treatment. As we believe, SPS sintering of the ZnO-In_2_O_3_ system in vacuum took place in a reducing atmosphere with the participation of reactive indium hemioxide due to the use of graphite tooling (punch and matrix of the press-form, sealing materials). This can explain the rapid formation of the new homologous In_2_Zn_5_O_8_ phase at the SPS temperature of 950 °C without registering an intermediate spinel ZnIn_2_O_4_ phase, as is usually observed in ZnO-Al_2_O_3_ and ZnO-Ga_2_O_3_ systems.

Thus, all synthesized ceramic samples had sufficient physical density and good electrical conductivity for their further use as targets in the formation of homogeneous thin films by dc magnetron sputtering [21]. It should be noted that all targets sputtered for more than 3 h retained their integrity (Figure S2 of SM). In this case, the formation of undesirable nodules in the erosion zone, which destabilize the sputtering process and affect the final properties of the deposited film, was found only in the target with a minimum content of indium impurities [22,23].

**Figure 2:**
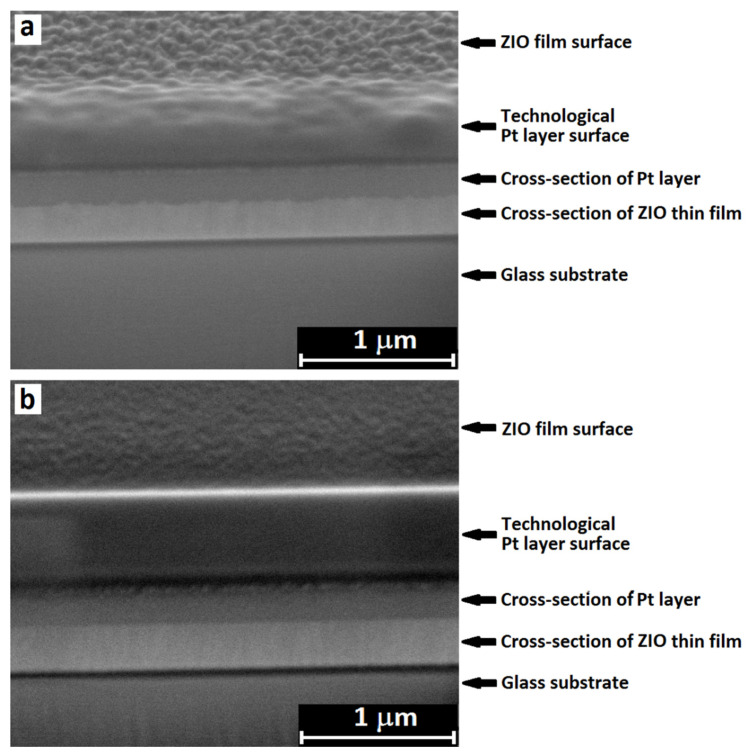
SEM micrographs of ZIO thin films deposited by using targets with In_2_O_3_ content of 0.5 (**a**) and 5.0 mol% (**b**). To obtain an image of transverse cleavage morphology, thin-film samples were subjected to local deposition of a technological platinum layer followed by ion etching.

Figure 2 shows SEM micrographs of ZIO thin films deposited by using the targets containing 0.5 mol% (which is below the solubility limit of In in ZnO of 4% [24]) and 5.0 mol% In_2_O_3_ (which is above the solubility limit). It can be seen that with an increase in the content of In_2_O_3_ in the target, the average grain size observed on the surface decreases and the film becomes smoother (this phenomenon is additionally demonstrated in Figure S3 in SM). The smooth surface morphology of TCO thin films is important for optoelectronic device applications. In addition, all deposited films are characterized by a columnar structure and with an increase in the In_2_O_3_ content, the average diameter of the columns decreases.

The results of evaluating the thickness *d* of ZIO thin films from SEM data showed that the growth rate of ZIO films increases monotonically with an increase in the impurity content (Table 1). Table 1 also shows the values of the In/Zn ratio for the films calculated from the EDX spectroscopy data (the corresponding EDX spectra and data are shown in Figure S4 in SM). It can be seen that, as a whole, the In/Zn ratio is in good agreement with the values initially set in the sputtered ceramics. The small deviations from the expected values observed here are most likely due to the insufficient set of statistical data during the thin film EDX measurements.

**Figure 3:**
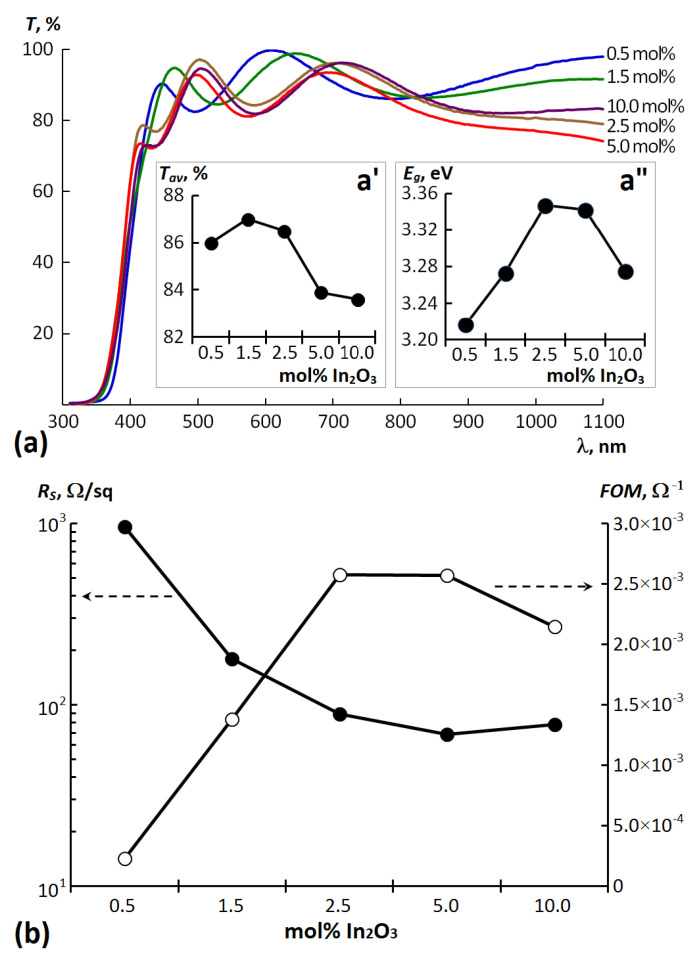
(**a**) Transmittance spectra in the 300–1100 nm region for ZIO thin films deposited by using the targets with various In_2_O_3_ content (Inset a’ and Inset a” show the dependences of the average transmittance *T_av_* and optical gap *Eg* of ZIO films on the In_2_O_3_ content, respectively); (**b**) Dependences of *R_S_* and *FOM* of the ZIO films on the In_2_O_3_ content in the sputtered ceramics.

The optical transmission spectra for the deposited ZIO thin films in the wavelength range between 380 and 1100 nm are shown in Figure 3a. It is seen that all films, despite the low deposition temperature, are characterized by a high optical transparency (>80%) in the visible range. Interference fringes caused by multiple reflections on different interfaces are observed in the spectra, which indicate that all deposited ZIO films are uniform and have smooth interfaces. The observed slight decrease in the distance between the interference fringes with increasing indium content indicates an increase in the optical path across the film, which is correlated with the SEM data regarding thefilm thickness.

With an increase in the doping level, the average transmittance *T_av_* in the visible region (380—760 nm) tends to decrease (see Inset a’ in Figure 3a). This may be due to an increase in the photon scattering on ZnO crystal lattice defects caused by the incorporation of In into the lattice [24,25,26].

Inset a” in Figure 3a shows the variations of the optical gap *E_g_* for the deposited ZIO films as a function of In_2_O_3_ content in the targets. The procedure for *E_g_* determining, which consists in extrapolating straight line portions of the Tauc’s graphs (α*h*ν)^2^ = f(*h*ν) to α*h*ν = 0 is demonstrated in Figure S5 in SM [27]. It is clear that the optical gap of the films is increased from 3.22 to 3.34 eV with an increase in In_2_O_3_ content from 0.5 to 2.5 mol%. A further increase in the content of indium oxide leads to a slight decrease in the optical gap to 3.26 eV. It is known that the magnitude of the shift correlates with the concentration of free charge carriers in a degenerate semiconductor [28]. Many authors reported band gap widening with an increase in donor concentration in ZnO, which is attributed to Burstein-Moss shift [29,30]. At the same time, the nonmonotonic nature of the change in the optical gap for the ZnO-Me_2_O_3_ system (Me = Al, Sc, Y and In), depending on the content of impurity oxide to the host ZnO, was observed by other authors [27,31]. The observed band gap narrowing in the ZIO films deposited from targets with In_2_O_3_ content above 2.5 mol% is associated with the formation of a band tail in the band gap with an increase in the carrier concentration [31,32], or, conversely, due to a possible decrease in the carrier concentration related with a high probability of the formation of charged acceptor complexes ($V_{Zn}^{2-}$– $In_{Zn}^{+}$) [33].

In confirmation of the above, as shown by measurements of electrical characteristics, the surface resistance *R_S_* decreases with an increase in the content of In_2_O_3_ to 5.0 mol%, and then slightly increases at 10 mol% (Figure 3b). The observed decrease in the sheet resistance of the films prepared in this work was due to In substitution into Zn sites (or interstitial sites). At the same time, the increase in *R_S_* at maximum In_2_O_3_ content may be explained by a combination of factors, including the loss of long-range crystal order [34], as well as an increase in impurity scattering due to the high concentration of charged dopant atoms in the film [12,13]. The thin film obtained by sputtering a target with 5.0 mol% In_2_O_3_ has a minimum sheet resistance *R_S_* of 69 Ω/sq and, accordingly, a maximum conductivity σ = 452.9 Ω^−1^·cm^−1^ (Table 1). A similar dependence of the conductivity on the doping level was previously observed for heavily In-doped ZnO films obtained by high-temperature dip-coating and aerosol assisted chemical vapour deposition methods [34,35]. They also noticed a decrease in conductivity at same doping level. In our case, it should also be noted that the achieved conductivity value for the ZIO thin film composition with 5.0 mol% In_2_O_3_ corresponds to one of the best among values declared for transparent electrodes formed on unheated substrates [36,37,38,39].

To evaluate the performance of transparent conductive films for various applications, the optical transmission and the electrical conduction of the films should be considered cumulatively. Usually, the objective evaluation can be carried out using Haacke’s figure of merit (FOM), defined as FOM = T^10^/R_S_, where *T* is the average visible transmittance [40]. The dependence of FOM of ZIO thin films on the level of doping with indium is shown in Figure 3b and in the last column of Table 2. For comparison, Table 2 also shows data on an ITO film with a thickness of 410 nm, deposited under completely identical technological conditions using a commercial ceramic In_2_O_3_:SnO_2_ (90:10 wt.%) target from Summit-Tech. Co. (Zhubei, Taiwan) [41]. It can be seen that for ZIO thin films, maximum FOM values 2.57 × 10^−3^ Ω^−1^ correspond to the ones deposited from ZnO targets with the In_2_O_3_ content of 2.5 and 5.0 mol%, while with further In_2_O_3_ content increasing the FOM starts to decrease. From the data presented in Table 2, it can be seen that these two ZIO films with the maximum FOM are only slightly inferior in their combination of optical and electrical properties to the traditional ITO material for transparent electrodes. However, an additional 20-min rapid thermal annealing (RTA) test of the deposited ZIO films at 200 °C in air revealed that the thermal stability of the films, defined as the ratio Δ*R*/*R_S_* (where Δ*R* is the difference between the sheet resistance after RTA R_RTA_ and the initial one *R_S_*) rapidly improves with increasing In_2_O_3_ content up to 10.0 mol%.

All these facts, taking also into account the obviousness of lower costs for a target of ZnO with 5.0 mol% In_2_O_3_ of compared to ITO one, makes it possible to consider this ZnO-based composition as a real alternative to ITO-based TCO films for various cases when a low-temperature deposition of transparent electrodes is needed.

## 4. Conclusions

In the presented work, the microstructure, phase composition, and physical properties of both SPS formed (ZnO–In_2_O_3_)-based ceramic targets and ZIO thin films deposited by following magnetron sputtering of these targets were studied. In particular, depending on the content of indium oxide in the sputtered target, the deposition process and the functional performances of the ZIO transparent conducting films at low substrate temperatures (≤50 °C) were studied in detail. It is shown that the In/Zn ratio in the ZIO films corresponds to the initial one, and with an increase in the In_2_O_3_ content in the sputtered target, the optical transmission coefficient of thin films tends to decrease monotonically to 83.6%, while the specific electrical conductivity reaches a maximum σ = 4.5 × 10^2^ Ω^−1^·cm^−1^ at 5.0 mol% In_2_O_3_. Thus, the ZIO film deposited from a target containing 5.0 mol% In_2_O_3_, having an average optical transmittance in the visible range of 84.1%, and a sufficiently thermostable electrical conductivity (4.5 × 10^2^ Ω^−1^cm^−1^), is characterized by the high *FOM* of 2.57 × 10^−3^ Ω^−1^, which is only slightly lower than the *FOM* value of a traditional ITO film deposited under the same conditions. From the point of view of commercial attractiveness, the obtained results make it possible to consider ZnO-based material with a composition of 95.0 mol% ZnO + 5.0 mol% In_2_O_3_ as an promising alternative to an expensive ITO in case of low-temperature formation of transparent electrodes, for example, in the flexible electronics area.

## Figures and Tables

**Table 1:** SEM and EDX data on film thickness *d* and in content for the deposited ZIO thin films.

Target	*d*, nm	In/Zn Ratio, %
99.5 mol% ZnO + 0.5 mol% In_2_O_3_	270 ± 5	1.1
98.5 mol% ZnO + 1.5 mol% In_2_O_3_	290 ± 5	2.9
97.5 mol% ZnO + 2.5 mol% In_2_O_3_	310 ± 5	4.6
95.0 mol% ZnO + 5.0 mol% In_2_O_3_	320 ± 5	9.2
90.0 mol% ZnO + 10.0 mol% In_2_O_3_	320 ± 5	26.1

**Table 2:** Measured sheet resistance *R_S_*, calculated film conductivity σ = (*R_S_* × *d*)^−1^, average transmittances *T_av_* in the range of 380–760 nm, and *FOM* of the ZIO and ITO thin films.

Target	*d*, nm	*R_S_*, Ω/sq	σ, Ω^−1.^cm^−1^	*T_av_*, %	*FOM*, Ω^−1^	Δ*R/R_S_*, *%*
99.5 mol% ZnO + 0.5 mol% In_2_O_3_	270 ± 5	960	38.6 ± 1.0	86.0	2.30 × 10^−4^	+132%
98.5 mol% ZnO + 1.5 mol% In_2_O_3_	290 ± 5	179	192.6 ± 3.5	87.0	1.38 × 10^−3^	+55%
97.5 mol% ZnO + 2.5 mol% In_2_O_3_	310 ± 5	89	362.5 ± 6.0	86.3	2.58 × 10^−3^	+37%
95.0 mol% ZnO + 5.0 mol% In_2_O_3_	320 ± 5	69	452.9 ± 7.0	84.1	2.57 × 10^−3^	+1%
90.0 mol% ZnO + 10.0 mol% In_2_O_3_	320 ± 5	78	400.6 ± 6.5	83.6	2.15 × 10^−3^	−4%
ITO [41]	410 ± 5	50	487.8 ± 6.0	82.6	2.96 × 10^−3^	–

## Supplementary Materials

The following are available online at https://www.mdpi.com/article/10.3390/ma14226859/s1, Figure S1: The XRD spectrum for the SPS-formed 90.0 mol% ZnO + 10.0 mol% In_2_O_3_ ceramic sample, Figure S2: A top view photography showing the appearance of ZnO-In_2_O_3_ targets subjected to more than 3 h of sputtering processes, Figure S3: SEM micrographs of top view showing the surface morphology of ZIO films deposited by using SPS synthesized ZnO-based ceramic targets with various In_2_O_3_ content, Figure S4: EDX spectra for ZIO thin films deposited by using the targets with various In_2_O_3_ content, Figure S5: Tauc’s graphs of ZIO thin films deposited by using the targets with various In_2_O_3_ content.
