# Supplementary material for: The ZnO-In2O3 Oxide System as a Material for Low-Temperature Deposition of Transparent Electrodes
Source: Materials (Basel). 2021 Nov 14;14(22):6859. doi: 10.3390/ma14226859 (PMC8618142; doi:10.3390/ma14226859)
Supplement: Supplementary file 1 [file materials-14-06859-s001.zip › materials-1448587-supplementary.pdf]

The ZnO-In_2O_3 oxide system as a material for low-temperature deposition of transparent electrodes

Akhmed Akhmedov [1], Aslan Abduev [2], Eldar Murliev [1], Abil Asvarov [3,*], Arsen Muslimov [3], Vladimir Kanevsky [3]

[1] Institute of Physics, Dagestan Federal Research Center, Russian Academy of Sciences, Makhachkala, Russia; cht-if-ran@mail.ru
[2] RUDN University, Moscow, Russia; a_abduev@mail.ru
[3] Shubnikov Institute of Crystallography, Federal Scientific Research Center Crystallography and Photonics, Russian Academy of Sciences, Moscow, Russia; amuslimov@mail.ru
* Correspondence: abil-as@list.ru; Tel.: +79303807650

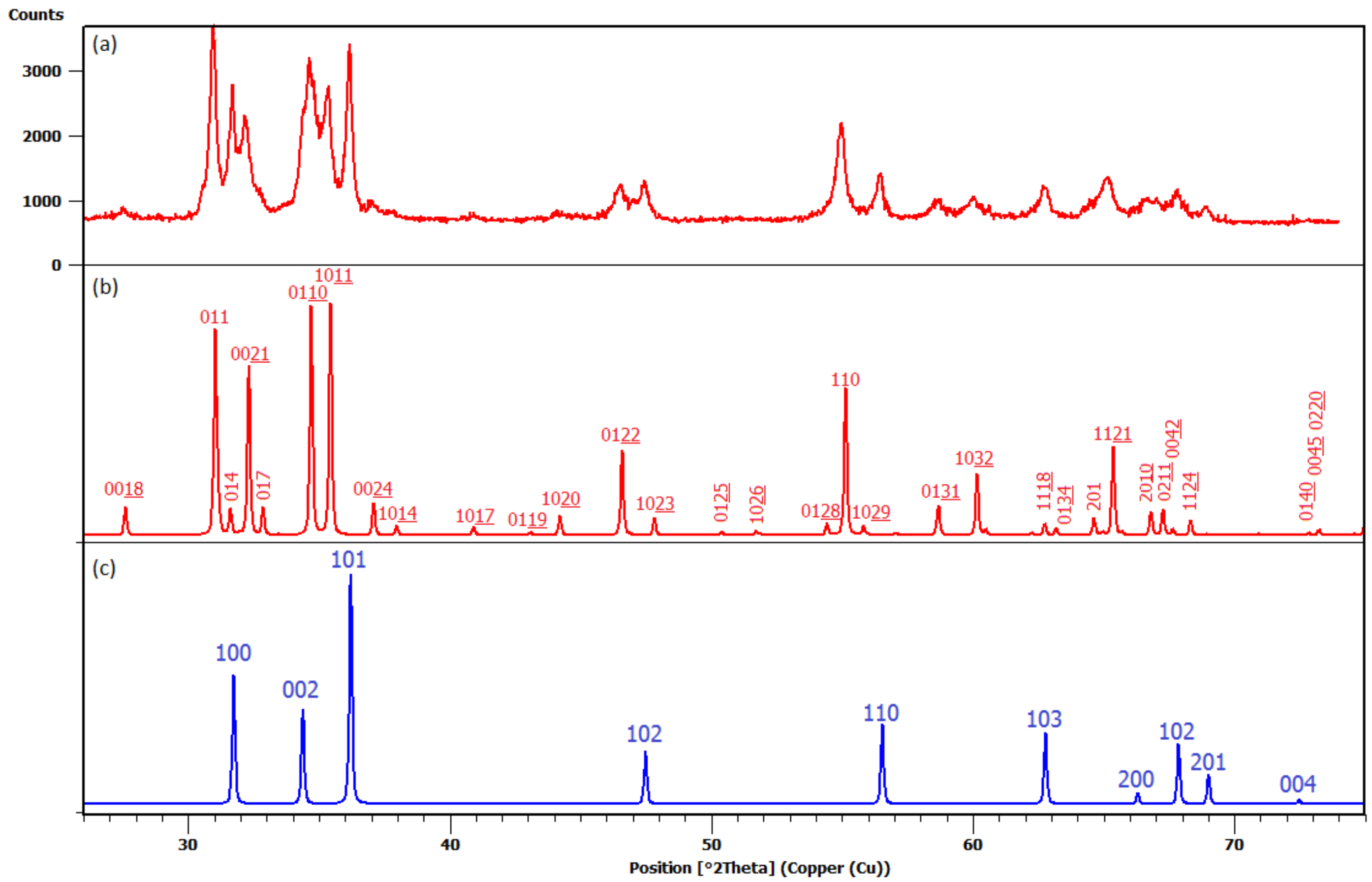

Figure S1. The experimental XRD spectrum for the SPS-formed 90.0 mol% ZnO + 10.0 mol% In_2O_3 ceramic sample (a); the simulated XRD spectrum for a well-crystallized $In_2Zn_5O_8$ hexagonal phase according to the JCPDS 01-089-8974 pattern card (b); the simulated XRD spectrum for a well-crystallized ZnO hexagonal phase according to the JCPDS 00-036-1451 pattern card (c).

The simulation procedure was performed by using the HighScore Plus software. The figure S1 clearly shows that the sintered ceramic sample contains two phases - ZnO and $In_2Zn_5O_8$.

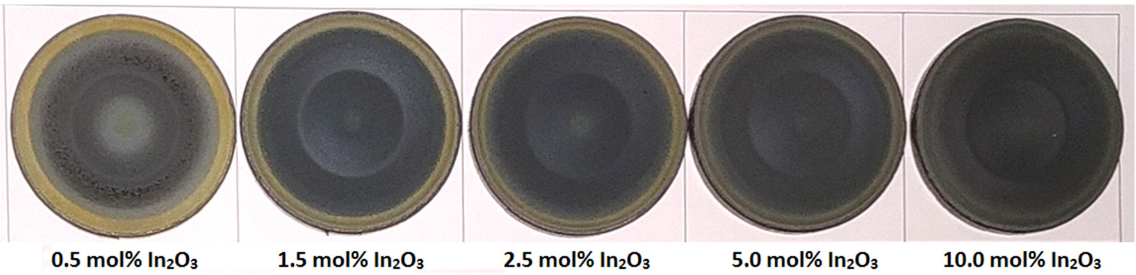

Figure S2. A top view photography showing the appearance of ZnO-$InO_{1.5}$ targets subjected to more than 3 hours of sputtering processes.

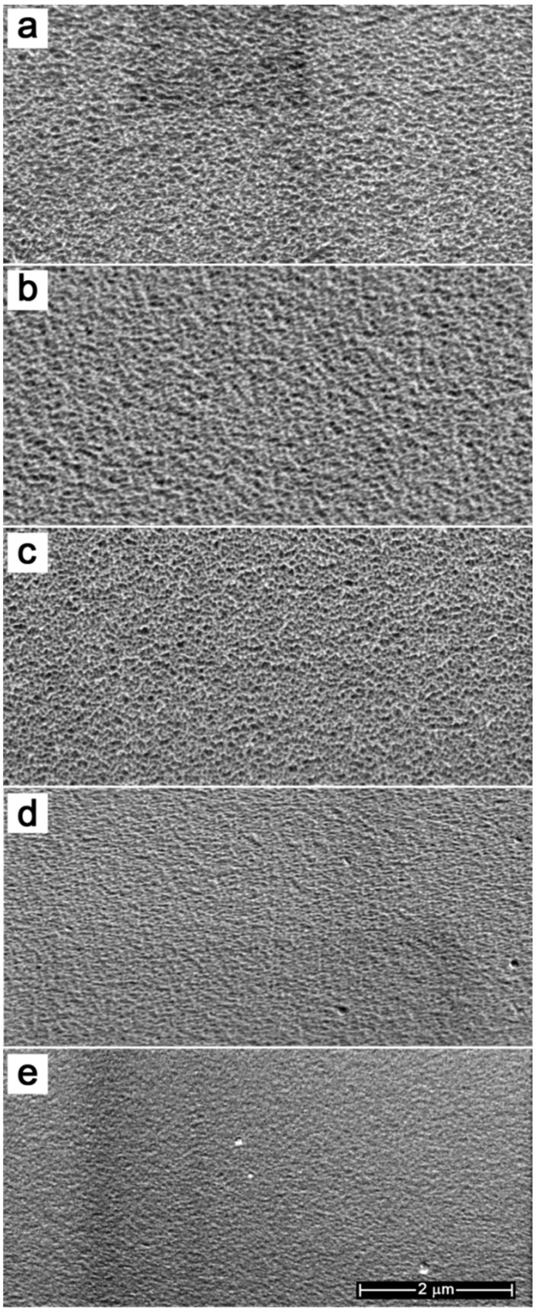

Figure S3. SEM micrographs of top view showing the surface morphology of ZIO films deposited by using SPS synthesized ceramic targets with various In_2O_3 content: a – 0.5 mol%, b – 1.5 mol%, c – 2.5 mol%, d – 5.0 mol% and e – 10.0 mol%.

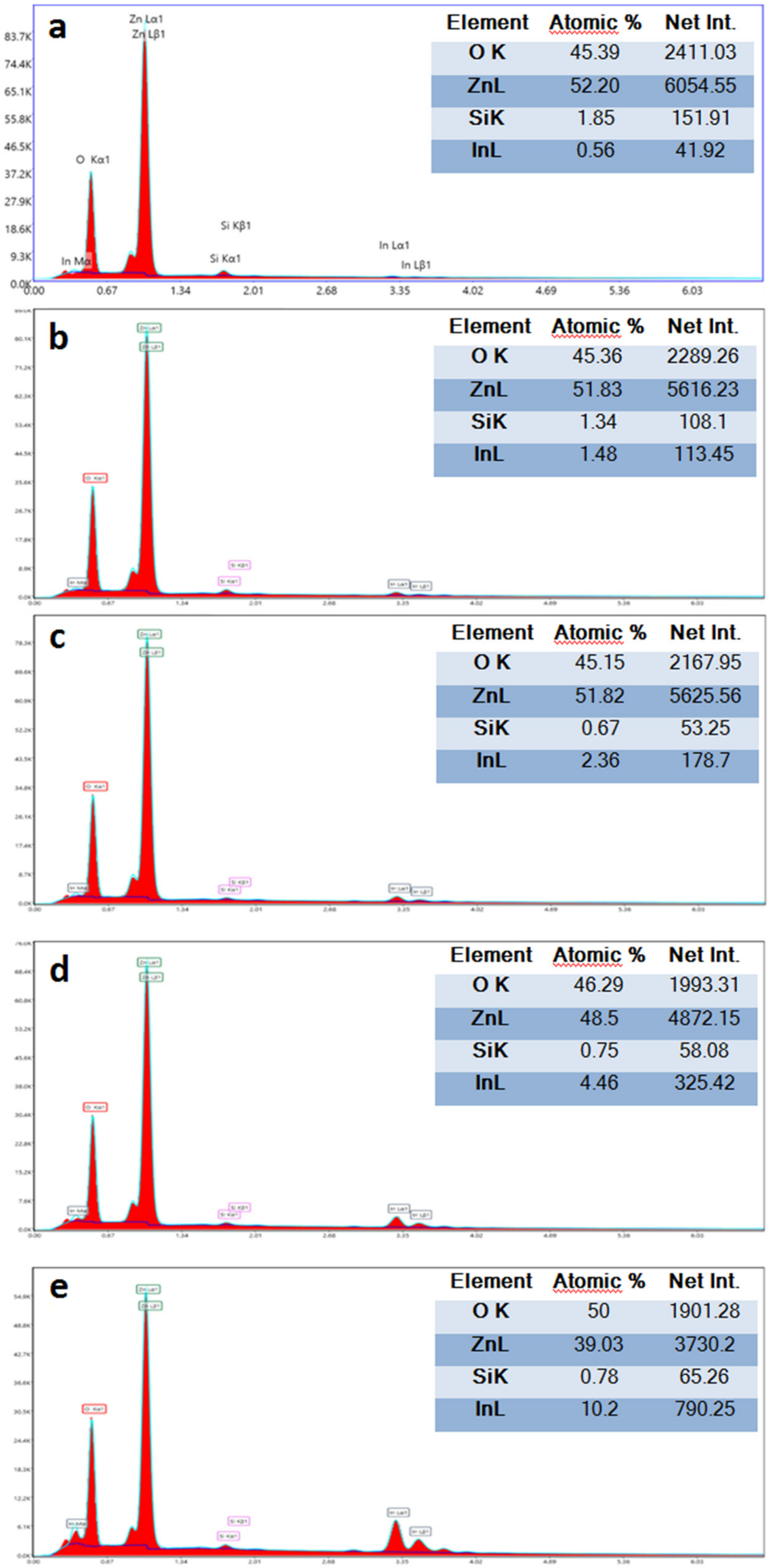

Figure S4. EDX spectra for ZIO thin films deposited by using the targets with various In_2O_3 content: a – 0.5 mol%, b – 1.5 mol%, c – 2.5 mol%, d – 5.0 mol% and e – 10.0 mol%. Insets show quantitative data on the elemental composition calculated by the eZAF Smart Quant method. The K-lines for Si present in the spectra came from the substrate.

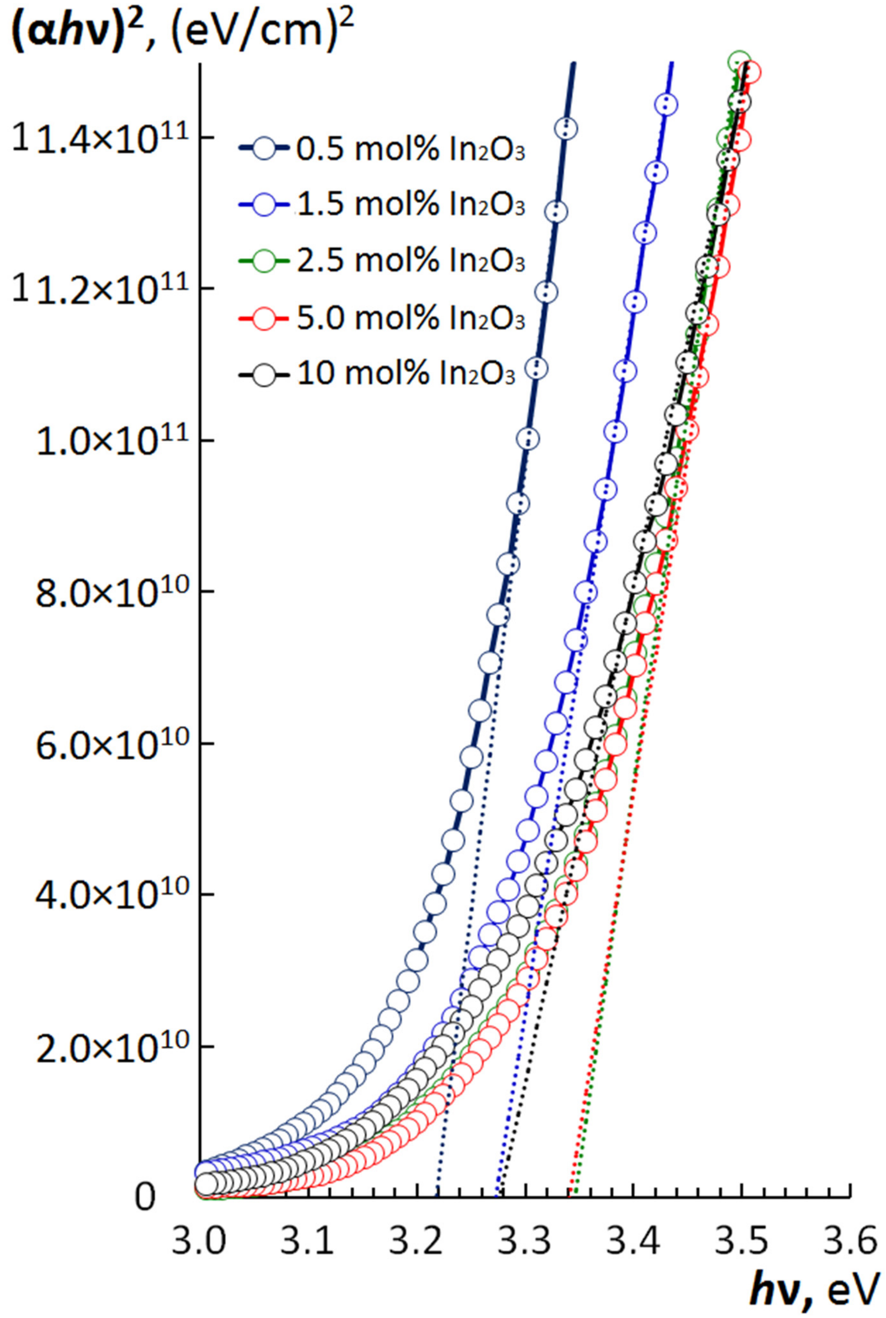

Figure S5. Tauc's graphs of ZIO thin films deposited by using the targets with various In_2O_3 content.
